# Does change in area-level deprivation, change health outcomes? A latent class growth analysis of population data

**DOI:** 10.1016/j.ssmph.2025.101826

**Published:** 2025-06-11

**Authors:** Finola Ferry, Ronald McDowell, Michael Rosato, Jamie Murphy, Gerard Leavey

**Affiliations:** a Bamford Centre for Mental Health and Wellbeing, Ulster University, Coleraine, Northern Ireland, BT52 1SA, UK; b Administrative Data Research Centre Northern Ireland (ADRC-NI), Dept of Psychology, Ulster University, Coleraine, Northern Ireland, BT52 1SA, UK

**Keywords:** Social mobility, Deprivation, Administrative data, Health, Mental health, Mortality

## Abstract

While deprivation is consistently predictive of health, it is typically studied at one point in time in relation to health outcomes. Emerging research indicates that trajectories of social mobility may be a more powerful predictor of health than point-in-time analyses. This study seeks to identify distinct area-level deprivation trajectories within the Northern Ireland (NI) population over multiple time-points and their associations with all-cause mortality; receipt of psychotropic medication; and presentations to Accident and Emergency (A&E) departments.

Based on linkage of NI GP registration, prescription, A&E and mortality data from 2010 to 2021, we used latent class growth analysis to identify trajectories in area-level deprivation from 2010 to 2016. Adjusting for baseline socio-demographic characteristics, we estimated the relationship between trajectories and health outcomes between 2017 and 2021.

We identified three stable, two downwardly mobile and two upwardly mobile classes. Upward mobility was associated with reduced risk of poor health outcomes compared to the consistently deprived. Downward mobility was associated with higher risk of poor health outcomes compared to the consistently non-deprived. An approximate dose-response relationship was observed across classes, whereby lower ‘endpoint’ deprivation in 2016 was associated with lower risk of adverse outcomes. The exception was the ‘substantial upward mobility’ class, with risk of poor outcomes second highest despite improved deprivation rank in 2016.

The classes of social mobility identified potentially provide a template within which social mobility can be studied in future research, highlighting the importance of both point of origin and destination in the study of social mobility and health.

## Highlights

• Application of data-driven method to study of social mobility using linked administrative data.
• End-point deprivation position associated with health outcomes, with one exception.
• Substantial upward mobility not associated with substantial reduction in risk of poor health.

## 1 Introduction

Trajectories of social mobility may be a more powerful predictor of health than point-in-time analyses (Islam & Jaffee, 2023; Jivraj et al., 2020). We attempted to examine social mobility and associated health outcomes in Northern Ireland (NI) using population-wide linked administrative data from 2010 to 2021.

Social Determinants of Health (SDH) are the non-medical factors that influence health outcomes, such as education, employment, housing and social class (Bell et al., 2010). Each of these are recognised as powerful determinants of health (Braveman & Gottlieb, 2014); SDH such as higher educational attainment (Raghupathi & Raghupathi, 2020), consistent employment (Pulford et al., 2022), and quality housing (Rolfe et al., 2020) are associated with more favourable health outcomes. The nature of the relationships between individual SDH and their combined effects on health outcomes is complex and varied. For example, being employed increases financial security, sense of self, relationships with others and social status (Pinto et al., 2018). It is estimated that together SDH can account for as much as 50 % of the variation in health outcomes (Hood et al., 2016). Poverty in its broadest sense (e.g. lack of financial income or access to resources such as health care, education and transport) is recognised as a major SDH which significantly affects health outcomes (American Association of Family Physicians, 2015). According to the 2022 Global Multidimensional Poverty Index (MPI), 19.1 % of the world's population are multidimensionally poor (UNDP, 2022).

At the neighbourhood level, impoverished areas affect the socioeconomic opportunities and outcomes of those who live in them (Charlton et al., 2013; Howell, 2019; Visser et al., 2021). Multiple deprivation measures (MDM) at area level have been used as proxies for SDH in previous work (Charlton et al., 2013; Skapinakis et al., 2005), comprising numerous domains of deprivation, including income, employment, education, housing, crime, and access to services. Furthermore, spatial measures of deprivation have been used to inform policy development and target areas of need in NI since the 1970s (Northern Ireland Statistics and Research Agency, 2010). Mental and physical health follow a social gradient, with studies consistently finding poorer health outcomes among those that live in deprived communities (Marmot, 2004, 2015). This finding is replicated when social disadvantage is measured by both individual-level SDH proxies such as education, employment, occupation, social class, and other indices; and when measured by area-level deprivation (Costa et al., 2002; Islam & Jaffee, 2023). NI has notably high levels of deprivation, where 37 % of the population live in the most deprived fifth of the UK (Abel et al., 2016). The most recent annual NI Health Inequalities Report indicates the persistence of wide-ranging health inequalities between the most and least deprived areas (Atcheson & Laverty, 2024). Social mobility is therefore a major goal of government policy to help ensure that the circumstances of birth do not determine outcomes in life (Social Mobility Commission, 2022).

Although neighbourhood deprivation is consistently associated with poor health, it is typically measured at one point in time. Indeed, evidence suggests that where deprivation exists, it persists; and the implications of living in deprivation are resistant to change (Raymond et al., 2024). Nevertheless, an individual's deprivation status can change, due to social mobility, the regeneration or decline of their neighbourhood, and important life events, among other influencing factors. A United Kingdom (UK) administrative data study (Fransham, 2019) noted a high flow of families in and out of deprived areas; while Jenkins (Jenkins, 2011) showed that annually 40 % of poor people leave, and 10 % of non-poor people enter poverty. Other recent reports indicate that the UK is characterised by *immobility* of those on low and high incomes as well as marked regional disparities (Social Mobility Commission, 2022; van der Erve et al., 2023). A ‘broad brush’ approach to the development of policies and interventions aimed at promoting social mobility is therefore unlikely to be effective. An enhanced understanding of social mobility profiles in different populations and how these are connected with health outcomes can potentially provide valuable evidence for the development of more targeted policies around social mobility and health.

While the last 25 years has seen a marked increase in the study of social mobility, our knowledge of social mobility and its relationship with health remains in its infancy. To date, the majority of studies have examined the association between deprivation and health utilising survey data and/or relatively small samples, either at one point in time or using a limited number of time points or social mobility periods. In a systematic review and meta-analysis of studies on social mobility and mental health, Islam and Jafee (Islam & Jaffee, 2023) concluded that both upwardly and downwardly mobile individuals have more mental health problems than those persistently advantaged and less than those persistently disadvantaged. While studies examining *intergenerational* social mobility and health can be identified (i.e. studies that define social mobility by comparing an individual's social class with parental social class) (Dal Grande et al., 2015; Dolan and Lordan; Tiikkaja et al., 2013), only a few longitudinal studies have examined *intragenerational* social mobility (based on indicators of social disadvantage among the same individual at different time points) and associated health outcomes. Similarly, few studies have formally compared health outcomes between the upwardly and downwardly mobile (Islam & Jaffee, 2023).

It has been suggested that the increased use of population administrative provides an opportunity to examine the relationship between deprivation and health outcomes in a more robust manner (Pattaro et al., 2020) The size and scope of such data, presents huge potential to develop a more nuanced understanding of real-world experience. There are only a few studies that have exploited this potential. Tseliou and colleagues (Tseliou et al., 2016), examining intercensal mobility (2001–2011) among children aged 0–8 years found that moving to a deprived area in childhood was linked to poorer mental health outcomes. Other administrative data studies report similar findings in relation to social mobility and mortality but are largely limited to comparison of socio-economic position (SEP) at two Census time points, or comparison of ‘family of origin’ and an individual's SEP (Blane et al., 1999; Dahl & Kjaersgaard, 1993; Hart et al., 1998; Schenk & Van Poppel, 2011).

The availability of population-level linked administrative data also offers fertile ground for the application of novel, data-driven methods to the study of social mobility and health. Data-driven research is an exploratory approach to analysing large datasets to extract scientifically interesting insights (Kitchin, 2014). In recent years these approaches have become an increasingly important tool for leveraging new insights from health data leading to enhanced patient care and improved outcomes (Batko & Ślęzak, 2022). Although these methods have been used to identify sub-groups of individuals with different trajectories of social mobility and their subsequent health outcomes these have been limited to relatively small cohort studies or surveys (Kwon et al., 2023; Logeswaran et al., 2023; Schiff et al., 2023). To the best of our knowledge, no such studies have been conducted using population-level linked administrative data. This longitudinal study therefore seeks to identify, for the first time, distinct trajectories in area-level deprivation (i.e. social mobility) within the NI population over multiple time-points and their associations with subsequent individual-level negative health outcomes, accounting for socio-demographic characteristics. Given that social mobility in NI has never been measured in this way, it was necessary to explicate and test both *a priori* and *post-hoc* hypotheses. The *a priori* hypotheses were based on the initial exploratory analyses. Specifically, we predicted that distinct social mobility groups (classes) of individuals would emerge from the data and would be characterised by either stability in deprivation status or change (i.e. upward/downward social mobility). Next, *post-hoc* hypotheses were generated and tested to determine the health consequences of social mobility. These hypotheses made health outcome predictions based on the unique social mobility class structure of the NI population identified in initial exploratory analyses.

## 2 Data & methods

### 2.1 Data sources and population

In NI the National Health Service (NHS) is free at the point of delivery, providing a wide range of primary, secondary and community-based care. We analysed linked administrative health data covering the years 2010–2021. The spine of the population was individuals registered with an NHS General Practitioner (GP) in NI in 2010. Virtually everyone in 10.13039/100013765NI registers with an 10.13039/100030827NHS 10.13039/100005129GP regardless of whether they access private or publicly funded services, thus ensuring a relatively comprehensive capture of the 10.13039/100013765NI population in 2010. Individuals were identified through the *GP Patient Registrations Index* and separately linked to other administrative datasets. These included the *Enhanced Prescribing Database* (EPD (which records all primary care prescriptions issued by community pharmacists and for which they received payment from the Health and Social Care Business Services Organisation (HSC BSO); and presentations to *Accident & Emergency* (A&E) departments, which were extracted from the *Northern Ireland Regional Accident & Emergency System* (NIRAES) and *Symphony* recording systems.

Area-level information on multiple deprivation and settlement urban-rural classification at baseline (2010) were provided by the Northern Ireland Statistical and Research Agency (NISRA) (Northern Ireland Statistics and Research Agency, 2010). Due to implementation of the Digital Economy Act in NI (2017), no legal gateway exists to link population-level health data and the ten-yearly population Census which includes information on other SDH such as education, employment and housing at the individual and household level. The month and year of all registered deaths were obtained from the *General Register Office for Northern Ireland* (GRONI). Those who emigrated during the study period (2010–2021) were excluded (as these individuals tend to be younger and healthier than resident populations) as well as those removed for reasons other than death (e.g.at their GP's request). All data were accessed securely via HSC NI Honest Broker Service (HBS), which operates under a memorandum of understanding: secondary data analysis of previously collected information for non-research purposes is exempt from Research Ethics Committee review, provided that patients or service users are not identifiable. Accredited researchers can only access the de-identified data used in this study after signing a Disclosure Policy Agreement and Research Data Access Agreement.

### 2.2 Exposure and covariates

The primary exposure variable for analysis was NIMDM-2010, the official measure of spatial deprivation (Northern Ireland Statistics and Research Agency, 2010) in NI. The NIMDM provides a mechanism for ranking of the 890 NI Super Output Areas (SOAS) from the most to the least deprived based on a weighted combination of seven domains of deprivation (income, employment, health deprivation and disability, education skills and training, proximity to services, living environment, crime and disorder), thus approximating numerous SDH at area level. More detailed information on the methodology and weighting process is available from the official NISRA report (Northern Ireland Statistics and Research Agency, 2010). Due to revisions in the NIMDM measure in 2017, the study period was divided into two parts (2010–2016 and 2017–2021), with area-level deprivation studied in first part of the study. NIMDM-2010 quintiles (1 = most deprived, 5 = least deprived) were obtained for each individual in the years 2010, 2012, 2014 and 2016. Confounding variables as recorded in 2010 included: age, sex and locale of residence (grouped as *urban -* cities or towns with a population of 5000 residents or more, or *rural -* populations of less than 5000 residents or open countryside).

### 2.3 Distal outcome variables (2017–2021)

The outcome variables were studied in the second part of the study period (2017–2021) subsequent to the exposure period. Three health outcomes were modelled in relation to prior trajectories in area-level deprivation: a) all-cause mortality; b) receipt of psychotropic medication - specifically hypnotics (British National Formulary (BNF) section 4.1.1), anxiolytics (BNF 4.1.2) and antidepressants (BNF 4.3); and c) presentation at A&E departments. Patients were followed up from January 1, 2017 until either death or December 31, 2021, whichever came first.

### 2.4 Statistical models

#### 2.4.1 Latent class growth analysis

Latent Class Growth Analysis (LCGA) is an exploratory analysis method which can be used with longitudinal data to identify subgroups of individuals (classes) whose growth trajectories with respect to a particular variable or variables over time are sufficiently similar to each other within each class, yet sufficiently different from other classes (Nagin, 2005). We therefore considered this an appropriate method to identify individuals with similar trajectories in area-level deprivation between 2010 and 2016 We hypothesised *a priori* that distinct social mobility classes would emerge and that these classes would be characterised by either stability in deprivation status or change (i.e. upward/downward mobility).

As the number of latent classes in the NI population was not known in advance, a series of LGCA were undertaken. The number of classes to be identified was increased by one for each iteration of the analysis and results were compared using various criteria. These included the Information Criterion (such as the Akaike Information Criterion (AIC), Bayesian Information Criterion (BIC) and Sample Size Adjusted-Bayesian Information Criterion (SSA-BIC) where lower values indicate better model fit (and hence a more preferable solution) (Wickrama et al., 2016); and entropy, a measure of class separation ranging from 0 to 1 with higher values indicating clearer class separation (Clark, 2010). However key considerations in identifying the optimal number of classes to be reported were ease of interpretation, real-world plausibility (i.e. solutions that captured the dynamic profile of social mobility – stable deprivation status; upward mobility; downward mobility) and the potential for new clinical insights (Geiser, 2013a; Wickrama et al., 2016).

NIMDM-2010 quintiles were treated as continuous rather than ordered categorical measures because LCGA analyses treating area-level deprivation as categorical estimate five probability trajectories (one per quintile) for each latent class thus making identification of the optimal solution challenging. The LCGA were estimated treating area-level deprivation as a continuous measure using Maximum Likelihood Robust (MLR) estimation, consistent with the literature (Benson & Fleishman, 1994; Rhemtulla et al., 2012).

Missing values of area-level deprivation were treated as non-informative and Missing at Random (MAR) (Mack et al., 2018). The LCGA analyses were estimated using all recorded area-level deprivation measures under Full Information Maximum Likelihood (FIML), without adjusting for baseline confounders, thus including all available neighbourhood deprivation data. Fuller details of LGCA and its application in this analysis are included in the Supplementary Materials Appendix A1.

#### 2.4.2 Associations between classes of social mobility and distal outcomes

To estimate associations between social mobility trajectories and subsequent outcomes, individuals were allocated to their most likely latent class on the basis of the posterior probabilities of class membership. This is appropriate when the degree of class separation (entropy) is high (>0.8) (Clark, 2010). Analyses were then conducted examining the association between social mobility class membership and distal outcomes, adjusting for baseline confounders.

Discrete-time survival analysis was used to estimate the hazard of death between 2017 and 2021. The underlying hazard was modelled using a restricted cubic spline as these are commonly used to model non-linear associations in regression models (Harrell et al., 1988). The proportional hazards (PH) assumption was examined by including interactions between each spline term and latent class, age, sex, locale of residence. Hurdle models (Cameron & Trivedi, 2013; Mullahy, 1986) were used to estimate associations between social mobility class membership and the other distal outcomes. This approach is commonly used to model health outcomes where there is excess zero counts in the data (Feng, 2021; Fernandez & Vatcheva, 2022). For each drug class the two-part model estimated the odds of receipt (using logistic regression) and, among those in receipt of the drug class, the number of prescriptions received per year (using a truncated negative binomial model). Analogous models were used for presentations to A & E.

Additional *post hoc* hypotheses were generated and tested to determine the health consequences of social mobility. Each hypothesis was tested relative to a corresponding null hypothesis, with a Bonferroni correction applied to control for the risk of a Type 1 error (false positive) due to multiple testing. All results were held to be significant if they refer to statistical significance on a 2-sided design-based test evaluated at the 5 % level.

### 2.5 Sensitivity analysis

The main analysis estimated the latent classes among individuals for whom outcomes were measured between 2017 and 2020. To determine the representativeness of our results the latent classes were re-estimated additionally including those who died between 2010 and 2016, but for whom, by definition, no outcomes post 2016 would be recorded.

### 2.6 Use of statistical software

The latent class analyses were run in Mplus 8.0 (Muthén & Muthén, 2017) with all other analyses conducted in Stata 18SE (StataCorp, 2023).

## 3 Results

### 3.1 Descriptive statistics

The main analysis included 1,569,110 individuals. These persons accounted for 95 % of the Northern Irish population registered with a GP in 2010 and who were living in NI in 2017, the year in which the distal outcomes were initially measured. Half of the population were male (50.1 % (n = 786,820)), the median age in 2010 was 36 years (InterQuartile Range (IQR) 19,52), with 3.6 % (n = 56,320) aged 75 years or over; 61.8 % (n = 968,284) lived in urban settings in 2010. Sixty percent (59.7 % (n = 922,915)) lived in areas with the same deprivation quintile in both 2010 and 2016, 19.3 % (n = 298,665) lived in a more deprived area in 2016 compared to 2010, and 20.9 % (323,736) lived in a less deprived area in 2016 compared to 2010. The NIMDM measures were recorded at four time-points for 98 % of individuals; the missingness pattern is detailed in Supplementary Table S1.

### 3.2 Latent class growth analysis solution

**Fig. 1:**
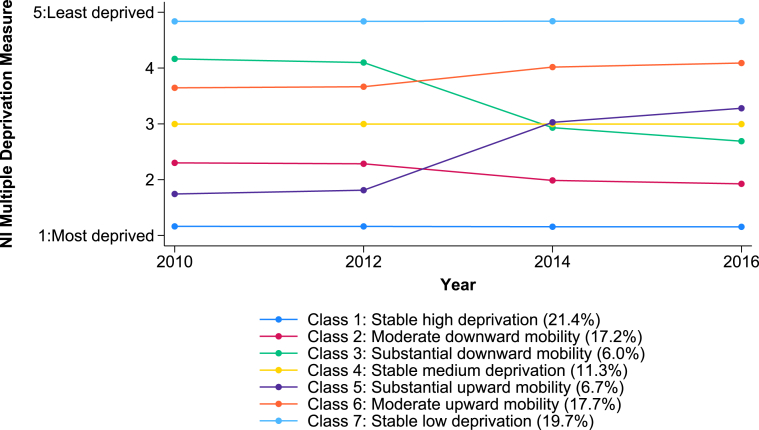
Estimated mean trajectories of area-level deprivation (2010–2016): 7 class Latent Class Growth Analysis (LCGA) solution.

Fit statistics for models between one and seven classes are reported in Table 1; beyond this the identified latent classes were less distinct, solutions were more difficult to interpret and any gain in model fit minimal. The seven-class LCGA solution was considered the optimal solution on the basis of superior model fit (Table 1) and real-world representation, as this solution allows for the possibility that individuals, could continue living in areas with similar levels of deprivation from 2010 to 2016, move into less deprived areas (i.e. upwardly mobile) or move into more deprived areas (downwardly mobile). No other LCGA solution with fewer than seven classes included all of these distinct trajectories. The average trajectories in area-level deprivation for each class are depicted in Fig. 1. The seven latent classes, enumerated from highest estimated area-level deprivation in 2016 (Class 1) to lowest (Class 7), are as follows.

**Table 1:** Fit statistics for non-linear Latent Class Growth Analysis (LCGA) models (n = 1,569,110).

Number of latent classes	Degrees of freedom	AIC	BIC	SSA-BIC	Lo-Mendell Rubin LRT Test	Entropy
1	8	21,757,985.2	21,758,083.3	21,758,057.9	–	1
2	11	17,131,629.3	17,131,764.2	17,131,729.2	p < 0.001	0.900
3	14	15,201,126.5	15,201,298.2	15,201,253.7	p < 0.001	0.946
4	17	14,478,247.2	14,478,455.8	14,478,401.7	p < 0.001	0.922
5	20	14,056,964.5	14,057,209.8	14,057,146.3	p < 0.001	0.941
6	23	13,691,279.8	13,691,561.9	13,691,488.9	p < 0.001	0.948
7	26	12,873,064.0	12,873,382.9	12,873,300.3	p < 0.001	0.965

AIC: Akaike Information Criterion; BIC: Bayesian Information Criterion; SSA-BIC Sample Size Adjusted Bayesian Information Criterion; LRT: Likelihood Ratio Test.

Three socially stable classes (52.4 %) were identified.

a) individuals consistently living in the most deprived areas (21.4 %) (*stable high deprivation*) (Class 1)
b) individuals consistently living in areas of average-level deprivation (11.3 %) (*stable medium deprivation*) (Class 4)
c) individuals consistently living in the least deprived areas (19.7 %) (*stable low deprivation*) (Class 7)

Two distinct groups of individuals (23.2 %) were downwardly mobile.

d) those living in slightly more deprived areas in 2016 compared to 2010 (17.2 %) (*moderate downward mobility*) Class (2).
e) those living in substantially more deprived areas in 2016 compared to 2010 (6.0 %) (*substantial downward mobility*) (Class 3).

Finally, two groups of individuals (24.4 %) were upwardly mobile.

f) those living in slightly less deprived areas in 2016 compared to 2010 (17.7 %) (*moderate upward mobility*) (Class 6)
g) those living in substantially less deprived areas in 2016 compared to 2010 (6.7 %) (*substantial upward mobility*) (Class 5).

The composition of each class according to sex, age-group and locale of residence is reported in Table 2. Individuals who consistently lived in the least deprived areas between 2010 and 2016 tended to be older than in the other social mobility classes (p < 0.001). Those who continually lived in either the most deprived or least deprived areas were predominantly urban dwellers (82.6 %, 72.5 % respectively) compared to the other classes (p < 0.001).

**Table 2:** Associations between social mobility class membership (2010–2016), socio-demographic characteristics and health outcomes (2017–2021).

	Class 1: Stable high deprivation	Class 2: Moderate downward mobility	Class 3: Substantial downward mobility	Class 4: Stable medium deprivation	Class 5: Substantial upward mobility	Class 6: Moderate upward mobility	Class 7: Stable low deprivation	p-value
**Overall**	21.4 % (336,147)	17.2 % (270,153)	6.0 % (94,366)	11.3 % (177,753)	6.7 % (104,273)	17.7 % (277,103)	19.7 % (309,315)	

**Socio-demographics**								
**Sex**								
Male	50.8 % (170,695)	50.5 % (136,290)	50.1 % (47,299)	50.4 % (89,603)	49.3 % (51,440)	50.0 % (138,458)	49.5 % (153,035)	p < 0.001
Female	49.2 % (165,452)	49.6 % (133,863)	49.9 % (47,067)	49.6 % (88,150)	50.7 % (52,833)	50.0 % (138,645)	50.5 % (156,280)	
**Age (years)**								
0-17	24.9 % (83,716)	24.0 % (64,902)	23.2 % (21,865)	24.0 % (42,635)	24.3 % (25,368)	23.4 % (64,922)	21.4 % (66,255)	p < 0.001
18-34	26.6 % (89,407)	24.8 % (67,008)	24.8 % (23,396)	24.2 % (43,018)	29.6 % (30,908)	23.1 % (63,880)	18.4 % (57,036)	
35-54	29.2 % (98,039)	29.7 % (80,196)	30.8 % (29,017)	29.7 % (52,856)	28.1 % (29,255)	31.3 % (86,661)	33.5 % (103,446)	
55-74	16.3 % (54,863)	18.0 % (48,649)	17.9 % (16,864)	18.5 % (32,939)	14.7 % (15,298)	18.6 % (51,609)	22.2 % (68,727)	
75+	3.0 % (10,122)	3.5 % (9398)	3.4 % (3224)	3.5 % (6283)	3.3 % (3434)	3.6 % (10,031)	4.5 % (13,851)	
**Locale of residence**								
Urban	82.6 % (274,581)	52.2 % (138,735)	42.9 % (40,269)	40.6 % (70,471)	72.3 % (75,082)	53.4 % (146,422)	72.5 % (222,724)	p < 0.001
Rural	17.5 % (58,038)	47.8 % (126,906)	57.1 % (53,574)	59.4 % (103,006)	27.7 % (28,726)	46.6 % (127,956)	27.5 % (84,453)	
**NIMDM quintile**^a^								
2010	1.2 (0.4)	2.3 (0.6)	4.1 (0.4)	3.0 (0.3)	1.8 (0.5)	3.6 (0.7)	4.8 (0.4)	p < 0.001

**Outcomes (2017–2021)**								
**All-cause mortality**	5.4 % (18,241)	5.1 % (13,743)	4.7 % (4442)	4.7 % (8409)	5.0 % (5263)	4.7 % (13,073)	4.9 % (15,185)	p < 0.001
**Antidepressants**	37.1 % (124,775)	32.4 % (87,613)	30.4 % (28,725)	29.4 % (52,332)	33.8 % (35,198)	28.9 % (79,997)	27.4 % (84,686)	p < 0.001
**Anxiolytics**	18.0 % (60,486)	15.3 % (41,442)	14.1 % (13,340)	13.8 % (24,566)	16.3 % (16,977)	13.9 % (38,459)	12.9 % (40,044)	p < 0.001
**Hypnotics**	14.4 % (48,264)	12.5 % (33,701)	11.4 % (10,723)	10.8 % (19,238)	12.6 % (13,083)	10.4 % (28,879)	10.1 % (31,339)	p < 0.001
**A&E visits**	63.5 % (213,489)	62.3 % (168,264)	61.1 % (57,698)	62.0 % (110,293)	63.5 % (66,213)	59.3 % (164,346)	56.8 % (175,723)	p < 0.001

A&E: Accident & Emergency; NIMDM: Northern Ireland Multiple Deprivation Measure.

a Mean (standard deviation).

### 3.3 *Post-hoc* hypotheses

On the basis of the 7-class social mobility structure identified above and existing literature, the following post-hoc hypotheses were generated.

Hypothesis a Given that area-level deprivation is associated with poor health outcomes, we hypothesised that individuals in the stable medium deprivation class (Class 4) and the stable low deprivation class (Class 7) would be less likely to experience negative health outcomes compared to the stable high deprivation class (Class 1). Effects would reveal a dose-response relationship.

Hypothesis b Both downwardly mobile classes would be more likely to experience negative health outcomes compared to the stable low deprivation class (Class 7). However, given that levels of deprivation in the moderate downward mobility class (Class 2) remained consistently higher than the substantial downward mobility class (Class 3), we hypothesised that the moderate downward mobility class would have poorer health outcomes compared to the substantial downward mobility class.

Hypothesis c Both upwardly mobile classes would be less likely to experience negative health outcomes compared to the stable high deprivation class (Class 1). However, because levels of deprivation in the substantial upward mobility class (Class 5) remained consistently higher than the moderate upward mobility class (Class 6), we hypothesised that the substantial upward mobility class would have poorer health outcomes compared to the moderate upward mobility class.

Hypothesis d Given that each of classes 3, 4 and 5 were characterised by low, moderate, and high area-level deprivation in 2010 and 2012 but converged to reflect moderate deprivation in 2014 and 2016, we tested the hypothesis that health outcomes differed across all three classes.

Each hypothesis was tested relative to a corresponding null hypothesis (see Supplementary Tables S4–S6).

### 3.4 Outcomes (2017–2021)

#### 3.4.1 All-cause mortality

**Fig. 2:**
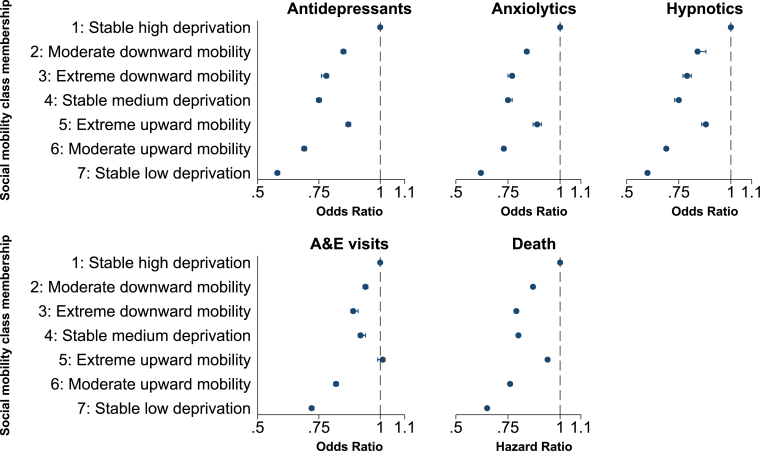
Forest plots: multivariable associations between social mobility class membership and outcomes (Odds Ratios).

Between 2017 and 2021 78,329 (5.0 %) individuals died. The proportional hazards assumption was not violated (p = 1.000). Compared to those who had continuously lived in the most deprived areas between 2010 and 2016, individuals from each social mobility class had a significantly lower risk of death after adjusting for age, sex and locale of residence (Fig. 2, full results in Supplementary Materials Table S2). A clear dose-response relationship was observed between area-level deprivation in 2016 and all-cause mortality, with the exception of the substantial upwardly mobile class.

Mortality increased with age, was lower for females (HR = 0.88: 0.87,0.89) and for those living in rural areas in 2010 (HR = 0.81: 0.80,0.83) (Table S2, Supplementary Materials Fig. S1).

#### 3.4.2 Receipt of psychotropic medicines

##### 3.4.2.1 Antidepressants

Antidepressants were the most commonly prescribed psychotropic medication between 2017 and 2021. Overall, 31.4 % received antidepressants at least once over this time (median = 19 prescriptions (IQR 4,42)). There was a statistically significant difference in receipt of antidepressants across the social mobility classes (p < 0.001), with the lowest levels (27.4 %) observed among individuals consistently living in the least deprived areas and the highest levels (37.4 %) observed among those continuously living in the most deprived areas (Table 2).

Compared to those who had continuously lived in the most deprived areas between 2010 and 2016, individuals from each social mobility class had a significantly lower odds of receiving antidepressants adjusting for age, sex and locale of residence (Fig. 2, full results in Supplementary Materials Table S3). As before a clear dose-response relationship was observed between area-level deprivation in 2016 and receipt of antidepressants, with the exception of the substantial upwardly mobile class.

Receipt was higher for females (OR 2.05: 2.03,2.06) lower in rural areas (OR 0.77:0.77,0.78) and peaked between the ages of 35–54 years after controlling for all other variables.

**Fig. 3:**
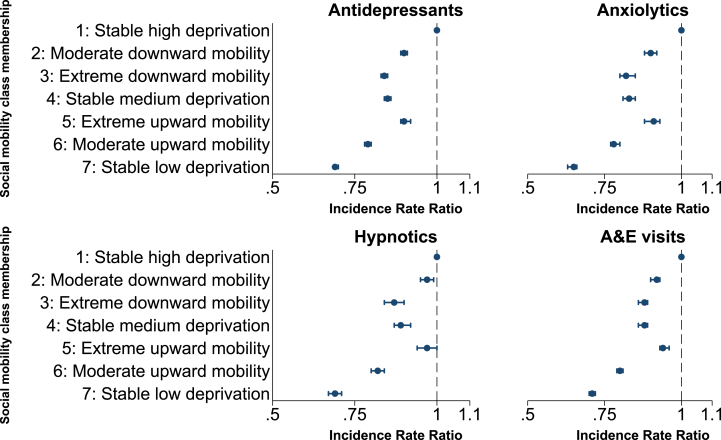
Forest plots: multivariable associations between social mobility class membership and outcomes (Rate Ratios).

Among individuals who received antidepressants, the relationship between social mobility class and the estimated number of antidepressants received per year was similar. For example, not only were those who consistently lived in the least deprived areas between 2010 and 2016 less likely to receive antidepressants than those who lived in the most deprived areas (OR 0.58: 0.58,0.59), but those who received them also received on average a lower number of items per year (IRR 0.69: 0.69,0.70). Full details are given in Fig. 3, Supplementary Materials Table S3.

##### 3.4.2.2 Anxiolytics and hypnotics

Overall, 15 % of the population were prescribed anxiolytics (median = 2, IQR = 1,10) and 12 % hypnotics (median = 4, IQR = 1,24) between 2017 and 2021. Levels of prescribing were lowest for those who continuously lived in the least deprived areas 2010–2016 (12.9 %, 10.1 % respectively) and highest for those who continuously lived in the most deprived areas (18.0 %, 14.4 % respectively).

Relationships between social mobility class membership, receipt of drug-type and number of items received per year replicated those observed for antidepressants (Fig. 2, Fig. 3, Supplementary Materials Table S3).

#### 3.4.3 Presentations at Accident & Emergency departments

Nearly two-thirds of individuals (60.9 %) attended A & E between 2017 and 2021 (median number of visits 2, IQR 1,4). Attendance differed significantly between social mobility classes (p < 0.001), with attendance lowest (56.8 %) among individuals who lived in the least deprived areas between 2010 and 2016 (Table 2).

The relationship between social mobility class and attendance at A&E followed a similar pattern to all-cause mortality and receipt of psychotropic medications. Compared to those who had continuously lived in the most deprived areas between 2010 and 2016, individuals from each social mobility class had a significantly lower odds of attending A&E after adjusting for age, sex and locale of residence, with the exception of the substantial upwardly mobile class (Fig. 2, Supplementary Materials Table S4). As before a clear dose-response relationship was observed between area-level deprivation in 2016 and A&E attendance, with the exception of the substantial upwardly mobile class. Attendance at A & E did not differ significantly by sex (OR 1.00: 0.99,1.00, p = 0.489) or locale of residence (OR 1.00: 0.99,1.01, p = 0.976) and was highest among those 75 years and over (Table S4).

The relationship between social mobility class and number of A & E presentations per year mirrored that of social mobility class and any A & E attendance. For example, not only were those who consistently lived in the least deprived areas between 2010 and 2016 less likely to attend A&E than those who lived in the most deprived areas (OR 0.72: 0.72,0.73), but if they attended, they attended less frequently (IRR 0.71: 0.70,0.72). Full details are given in Fig. 3, Supplementary Materials Table S4.

#### 3.4.4 Post-hoc hypotheses testing

hypothesis a This hypothesis was accepted for each outcome (p < 0.001). Estimated outcomes were poorest in the stable low deprivation class (Class 7), were better for those in the stable medium deprivation class (Class 4) and were best in stable low deprivation class (Class 7). This dose-response relationship was observed for each outcome. For example, the estimated risk of death was 20 % lower (HR 0.80: 0.79,0.80) for those who had continuously lived in areas of average-level deprivation and around a third lower (HR 0.65: 0.64,0.65) for those who had continuously lived in the least-deprived areas compared to those who continuously lived in the most deprived areas.

hypothesis b This hypothesis was accepted for each outcome (p < 0.001), with both downwardly mobile classes more likely to experience negative health outcomes compared to the stable low deprivation class (Class 7). The moderate downward mobility class (Class 2) had poorer health outcomes compared to the substantial downward mobility class (Class 3). For example, receipt of antidepressants was lowest in the stable low deprivation class (OR 0.58 (0.58,0.59). On average the odds of receiving antidepressants were 9 % higher for individuals who experienced a small decline in social mobility compared to those who had experienced a substantial decline (OR 1.09: 1.07,1.11)

hypothesis c This hypothesis was accepted for each outcome (p < 0.001). Both upwardly mobile classes were less likely to experience negative health outcomes compared to the stable high deprivation class (Class 1). The substantial upward mobility class (Class 5) had poorer health outcomes compared to the moderate upward mobility class (Class 6). For example, the odds of presenting at A &E was highest for those in the stable high deprivation class (OR 1.00). On average A&E attendance was 22 % higher for individuals who experienced a small decline in social mobility when compared to those who had experienced a substantial decline (OR 1.22: 1.21,1.24)).

hypothesis d This hypothesis was accepted for each outcome (p < 0.001). Health outcomes differed across each of for each of the Classes (3,4,5) who converged to converged to reflect moderate deprivation in 2014 and 2016. For example, the risk of death between 2017 and 2021 differed significantly (p < 0.001) between each of these classes and was highest for those with a substantial improvement in social mobility (HR 0.94 (0.94,0.95). The risk of death was similar in the other two classes (Class 3: HR 0.79 (0.78,0.79); Class 4: HR 0.80 (0.79,0.80)

#### 3.4.5 Sensitivity analysis

The LCGA models were re-estimated to include individuals who died between 2010 and 2016 and hence for whom outcomes could not be observed after 2016. In this analysis area-level deprivation was recorded for 93 % of individuals at each time-point and for 95.4 % of persons at least three times. The seven-class solution was again identified as the optimal solution, with mean trajectories and class sizes virtually identical to the main analysis (Supplementary Materials Fig. S3). For the 1,569,110 individuals alive at the start of 2017, 1,566,672 (99.8 %) were allocated to the same latent class as in the main analysis hence relationships between latent classes and outcomes were identical to the main analysis.

## 4 Discussion

### 4.1 Principal findings

This is the first linked administrative data study to use a data-driven technique to identify trajectories in area-level deprivation over time at the population level; and examine their associations with a range of subsequent health outcomes. Our analyses identified seven distinct classes. Around half of the population belonged to three classes characterised by deprivation stability (low, medium and high deprivation); two classes were downwardly mobile (moderate decline, more severe decline); two were upwardly mobile (moderate improvement, substantial improvement). Each social mobility class had better health outcomes between 2017 and 2021 than the stable high deprivation class after adjusting for age, sex and locale of residence. The risk of each health outcome was lowest for those consistently living in the least deprived areas between 2010 and 2016, higher for those living in consistently areas, and highest for those living in the most deprived areas. Overall, with the exception of the substantial upward mobility group (class 5), findings showed a dose-response relationship, whereby lower ‘end-point’ deprivation in 2016 was associated with lower risk of adverse health outcomes. The downwardly mobile were more likely, and the upwardly mobile less likely, to experience adverse health outcomes compared to the stable low deprivation class and high deprivation class, respectively. Those who experienced substantial upward mobility from a relatively deprived neighbourhood had poorer outcomes than those experiencing a small improvement in upward mobility but who had lived in less deprived areas. Health outcomes differed significantly among all three classes of individuals living in areas of average-level deprivation in 2016. These trends were replicated when examining yearly rates of psychotropic medication receipt or A & E attendance.

### 4.2 Findings within the context of other studies

Few studies have used data-driven techniques to measure and profile social mobility or trajectories of deprivation (Cundiff et al., 2017; Kwon et al., 2023; Logeswaran et al., 2023; Schiff et al., 2023; Sturgis & Sullivan, 2008; Yamaguchi, 2009), and the number of distinct trajectories identified in these studies has varied (6 classes (Kwon et al., 2023; Logeswaran et al., 2023); 5 classes (Sturgis & Sullivan, 2008); 4 classes (Schiff et al., 2023)). To our knowledge, this study is the first to employ a data-driven approach to model social mobility using population-wide linked administrative data. Moreover, the seven-class model we have identified, not only captures upward and downward mobility, but (unlike most previous studies) differentiates between mobility from different points of origin on the deprivation spectrum, which, it seems, has an important bearing on all the health outcomes we considered.

Our findings in relation to the three stable social mobility classes showing greatest risk of all four health outcomes in stable high deprivation and lowest risk of prescribing for those in stable low deprivation, and a dose response effect across all three stable classes are consistent with previous evidence on social mobility, mental health and mortality (Dal Grande et al., 2015; Ericsson et al., 2019; Nilsson et al., 2005). While numerous studies have evidenced the relationship between A&E attendances and psychotropic medication with area-level deprivation (Elwadhi & Cohen, 2020; Gethings et al., 2023; Scantlebury et al., 2015), no studies could be identified that have examined *trajectories of social mobility* in relation to these outcomes. Our paper therefore provides valuable new insights in this respect.

Our findings in relation to the socially mobile are largely consistent with evidence from other studies showing a reduction in risk of adverse health outcomes among those who are upwardly mobile, but an increase in risk among the downwardly mobile (Islam & Jaffee, 2023; Logeswaran et al., 2023; Tseliou et al., 2016). For example, Tseliou and colleagues found that moving to a deprived area in childhood was associated with poorer mental health among young people (Tseliou et al., 2016), while Logeswaren and colleagues found that upward mobility mitigated the association between childhood exposure to deprivation and risk of serious mental illness (Logeswaran et al., 2023). While upward mobility in general was associated with lower risk of poor outcomes, health outcome gains were minimal for the substantially upwardly mobile, whose point of origin was relative deprivation. Risk of adverse outcomes among this group were second highest only to the persistently deprived. There may be multiple reasons for this phenomenon. First, there may lingering consequences of prior SES, potentially reflecting lasting changes to stress response systems and emotional processing; and a sense of not belonging to the culture of a new social class (Islam & Jaffee, 2023). Economic disadvantage has been shown to increase the risk of poor health outcomes later in life, such as depression (Almeida et al., 2012) with some of the poor health conditions associated with deprivation likely to be maintained across the life-course. Cumulative stress, or the accumulation of stressors over time, is linked with a wide range of negative health outcomes (Haight et al., 2023). Second, it has been argued that upward mobility may also confer social and health penalties created by the loss of old social networks and the psychological challenges of ‘fitting in’. Thus, while health improvement in the upwardly mobile is often a modest gain, many individuals from low SES also carry experiences and a habitus (Bourdieu, 1977) very different to people whose advantaged situation has remained stable (Dinos, 2015).

A key finding from our analysis relates to risk of poor health outcomes among the three classes which converged around average level deprivation by 2016. Few studies have tested whether upwardly and downwardly mobile individuals differ from each-other with respect to health outcomes (Islam & Jaffee, 2023). We found significant differences between these classes in terms of the risk of death, receipt of psychotropic medication and A/E attendance between 2017 and 2021, with risk highest in the substantially upwardly mobile class for each outcome. These individuals, whose point of origin was relative deprivation in 2010 experienced a significant but relatively small reduction in risk despite substantial upward mobility. The effects associated with the two other classes, the stable medium deprivation class and the substantial downwardly mobile classes on each health outcome were similar in magnitude. Similar patterns are reported by Miething and colleagues, who found that health outcomes responded to lowered rank position in income to a greater degree than income gains over time (Miething & Åberg, 2014). Our findings point to the importance of point of origin and point of destination on the deprivation spectrum for some upwardly and downwardly mobile groups, respectively; and suggest that the residual association of having lived in areas of deprivation may have an important bearing on health, despite improvement in SEP.

### 4.3 Strengths and limitations of study

Our findings should be interpreted with a number of limitations in mind. First, both social mobility trajectories and subsequent health outcomes were followed up over relatively short time frames. While the tracking of social mobility over a longer time frame would have been preferable, changes to official NIMDM in 2017 meant that MDM (2010 version) data were not available beyond 2016 for inclusion in the current study. Second, the issue of causality is important. While improvement or deterioration in social circumstances can lead to changes in health, health may influence social circumstances. For example, deteriorating health may impact through loss of income and other resources. Third, the NIMDM provides an overall measure of area-level multiple deprivation based on a weighted combination of seven domains of deprivation, inclusive of a health domain, which constitutes 15 % of the overall score. Despite the risk of endogeneity bias, a recent Scottish linked administrative data study exploring potential bias effects found that the risk of endogeneity in health inequalities analysis was minimal, with similar results yielded by excluding the health domain from the overall measure or using only the income domain (Bradford et al., 2023). Fourth, we only tested a limited number of hypotheses in relation to social mobility classes and health outcomes. Fifth, the large sample size results in a highly powered study with multiple effects reaching formal statistical significance which may not all be of clinical or substantive significance. Finally, due to current NI legislation, it was not possible to include other individual-level SDH proxies in our analyses. This limited our ability to include other, more specific SDH and study their relationships to each other, to social mobility, and their combined effect on health outcomes.

Notwithstanding these limitations, our study has notable strengths. Firstly, the application of a novel analytical approach to the analysis of population-based administrative data extends previous applications of data-driven methods limited to survey or cohort studies and has afforded the identification of distinct social mobility trajectories among the overall population. Given the regionally disparities across the UK with respect to social mobility alluded to in recent reports (Social Mobility Commission, 2022; van der Erve et al., 2023), our study provides timely evidence that will help inform NI-specific, as opposed to UK-wide policies. Secondly, it was notable that a population-level data analysis revealed social mobility in a much more nuanced and detailed way. While previous studies have attempted to distinctly (and manually) classify and differentiate between upwardly and downwardly mobile groups, our population-based profiles clearly evidenced that multiple discrete groups of socially mobile individuals are not only characterised by different starting and end positions, but severity gradients also. Finally, the linkage of multiple administrative datasets over time has provided the opportunity to build on the evidence base in relation to *intragenerational* social mobility. Linkage of these health datasets has enabled an examination of multiple health outcomes, including A&E attendance which has been sparsely studied in relation to social mobility.

### 4.4 Implications

The recent ‘State of the Nation’ report and subsequent policy framework by the UK Social Mobility Commission (Social Mobility Commission, 2022; Social Mobility Commission. Innovation Generation, 2024) identified marked regional disparities in social mobility trends and their drivers, with NI ranked lowest for ‘promising prospects’, lagging behind other regions in terms opportunities and quality in education and employment. The reports thus highlight the necessity for ‘placed-based’ policies and interventions led by devolved governments and the need for more reliable data and analysis as an evidence-base for policy. Our study represents a step in the right direction in providing NI specific, population-based evidence on social mobility trajectories and a range of associated health outcomes that can inform the development of these more targeted devolved policies aimed at improving public health and addressing health inequalities.

Our findings highlight that the relationships between deprivation, social mobility and health are demonstrably complex and non-linear. Notwithstanding this complexity, our study confirms the relative benefits of upward mobility per se across all health outcomes considered. Given health inequalities between the most and least deprived areas reported here and elsewhere (Atcheson & Laverty, 2024), and with just 7 % upwardly mobile from the most deprived areas, our study provides further rationale for the development of targeted policies and initiatives that promote social mobility in the most deprived areas, such as investment in education and work prospects, which lag behind other UK regions (Social Mobility Commission, 2022). Our findings also support the development of necessary actions outlined in the 10.13039/100013765NI Mental 10.13039/100018696Health Strategy 2021–31 (Department of Health NI, 2021), such as campaigns that raise awareness and promote public discourse around mental health and its social determinants; increasing the supports to individuals, families and communities, particularly in deprived areas; and expansion of community-based services and supports such as therapy hubs.

We found that upward mobility does not always translate into substantially better health, with modest health gains for those upwardly mobile from the most deprived areas. The reversal or mitigation of the impacts produced by living in deprived areas through upward mobility may be harder to achieve than policymakers would wish. Our findings of the potential residual effects of having lived in deprivation underlines the importance of ‘front-loading’ of resources as early in the life-course as possible; and provide clinicians with valuable evidence on the importance of previous as well as current social context that can inform conversations and treatments with patients. With legislative changes in NI it will be possible to study in greater detail the relationship between social mobility, multiple SDH and health outcomes.

## 5 Conclusion

Social mobility and social immobility are present in NI, with those who continuously live in the most deprived areas having the poorest health outcomes. Upward mobility in itself does not necessarily ameliorate this phenomenon. Government policies aimed at improving health outcomes at the population level should prioritise early intervention and investment in both healthcare resources and development of education and work prospects in deprived communities. While current social status has an important bearing on health, patient-centred treatments should also be informed by previous social circumstances.

## CRediT authorship contribution statement

**Finola Ferry:** Writing – original draft, Project administration, Methodology, Formal analysis. **Ronald McDowell:** Writing – original draft, Validation, Methodology, Formal analysis. **Michael Rosato:** Writing – review & editing, Supervision, Project administration, Data curation. **Jamie Murphy:** Writing – review & editing, Validation, Supervision, Methodology. **Gerard Leavey:** Writing – review & editing, Supervision, Project administration, Funding acquisition, Conceptualization.

## Ethics statement

Secondary data analysis of previously collected information for non-research purposes is exempt from Research Ethics Committee review, provided that patients or service users are not identifiable. Ethical approval was facilitated via the Health and Social Care Northern Ireland (HSCNI) Honest Broker Service (HBS) which has a Memorandum of Understanding in place to permit usage of HSCNI datasets for research purposes. The HBS is the only resource allowing linkage of health-related data sources at the population level. Accredited researchers can only access the de-identified data used in this study after signing a Disclosure Policy Agreement and Research Data Access Agreement. Data is accessible by researchers only from a physically *secure setting* managed by the data custodians.

## Funding

This work was undertaken for a project as part of Administrative Data Research Northern Ireland, which is supported by the 10.13039/501100000269Economic and Social Research Council (10.13039/501100000269ESRC) (project number: ES/L007509/1).

## Declaration of competing interest

The authors declare that they have no known competing financial interests or personal relationships that could have appeared to influence the work reported in this paper.

## Data Availability

The authors do not have permission to share data.
