# Supplementary material for: Does change in area-level deprivation, change health outcomes? A latent class growth analysis of population data
Source: SSM Popul Health. 2025 Jun 11;31:101826. doi: 10.1016/j.ssmph.2025.101826 (PMC12221537; doi:10.1016/j.ssmph.2025.101826)
Supplement: Multimedia component 1 [file mmc1.docx]

**Supplementary Materials**

**Appendices**

Appendix A1 Latent Class Growth Analysis

**Tables**

Table S1 Missingness pattern of NI Multiple Deprivation Measure (2010-2016)

(n=1,569,110)

Table S2 Multivariable associations between social mobility class membership (2010-2016) and death (2017-2021)

Table S3 Multivariable associations between social mobility class membership (2010-16) and receipt of psychotropic medication (2017-21). Coefficients represent a) receipt (Odds Ratios) b) rate of receipt (Incidence Rate Ratios)

Table S4 Multivariable associations between social mobility class membership (2010-2016) and presentations to A&E departments (2017-21) Coefficients represent a) attendance (Odds Ratios) b) rate of attendance (Incidence Rate Ratios)

Table S5 Post hoc hypothesis testing of the associations between social mobility class membership (2010-2016) and death (2017-2021)

Table S6 Post hoc hypothesis testing of the associations between social mobility membership (2010-2016) and receipt of antidepressants (2017-2021)

Table S7 Post hoc hypothesis testing of the associations between mobility class membership (2010-2016) and presentations to A&E departments (2017-2021)

**Figures**

Figure S1 Multivariable associations between sociodemographic variables and health outcomes (Odds Ratios)

Figure S2 Multivariable associations between sociodemographic variables and health outcomes (Incidence Rate Ratios)

Fig S3 Estimated mean trajectories of area-level deprivation (2010-2016): 7 class Latent Class Growth Analysis (LCGA) solution (n=1,669,898)

**Appendix A1 Latent Class Growth Analysis**

We used Latent Class Growth Analysis (LCGA) to identify groups of individuals (classes) following similar trajectories in area-level deprivation between 2010 and 2016. Latent Class Growth Analysis (LCGA) is a data-driven method which identifies distinct subgroups of individuals, whose growth trajectories are sufficiently similar to each other within each class, yet sufficiently distinct from other classes [1] . It is a simplified form of Growth Mixture Modelling (GMM) [2] where the within-class variation is fixed at zero. Analyses conducted using GMM yielded improper solutions (negative variances) [3]so the simpler LCGA models were estimated, an approach consistent with the literature [4]. Quintiles of multiple deprivation were treated as a continuous measure because LCGA analyses treating area-level deprivation as categorical estimate five probability trajectories (one per quintile) for each latent class thus making identification of the optimal solution challenging. The LCGA were thus estimated treating area-level deprivation as a continuous measure using Maximum Likelihood Robust (MLR) estimation, an approach consistent with the literature[5], [6] .

Conventional model fitting practices were used to identify the optimum number of latent classes. Initially a one class solution was fit to the data. The number of classes was increased incrementally in steps of one and the LCGA model refitted until this was no longer computationally feasible. Sufficient random starts were used for each model to ensure that a global solution had been obtained. The models with different numbers of classes were then compared in terms of model fit using statistics such as the Information Criterion, Entropy and the Lo-Mendell-Rubin Likelihood Ratio Test [7]. Lower values of the Information Criterion (such as the Akaike Information Criterion (AIC), Bayesian Information Criterion (BIC) and Sample Size Adjusted-Bayesian Information Criterion (SSA-BIC) indicated comparatively better model fit. Entropy is a measure of class separation; it is reported as a standardised index (ranging from 0 to 1) with higher values indicating clearer class separation [8]. The Lo-Mendell-Rubin Likelihood Ratio Test formally compares a given latent class solution with that in which there is one fewer class. The Bootstrapped Likelihood Ratio Test [9] was not used for the evaluation of model fit as this led to convergence problems. Missing values of NIMDM2010 were considered to be non-informative and Missing At Random (MAR) i.e. any systematic differences between observed and missing values of area-level deprivation can be explained by other variables in the data [10]. Models were estimated using all available data under Full Information Maximum Likelihood (FIML). All LCGA models were fitted without adjusting for any baseline confounders.

The optimum number of classes was determined on the basis of conventional model-fitting practices, interpretability and substantive interest [4,11].

**Table S1: Missingness patterns for NIMDM measures (n=1,569,110)**

| **NIMDM 2010** | **NIMDM 2012** | **NIMDM 2014** | **NIMDM 2016** | **No of individuals** |
| --- | --- | --- | --- | --- |
| X | X | X | X | 1,537,815 |
| X | X |  |  | 7,671 |
| X |  |  |  | 5,340 |
| X | X | X |  | 5,039 |
| X |  | X | X | 4,192 |
|  |  | X | X | 2,647 |
| X | X |  | X | 2,172 |
|  | X | X | X | 1,626 |
|  |  |  | X | 1,344 |
| X |  |  | X | 1,137 |
| X |  | X |  | 63 |
|  | X |  |  | 20 |
|  |  | X |  | 18 |
|  | X | X |  | 16 |
|  | X |  | X | 10 |

NIMDM: Northern Ireland Multiple Deprivation Measure (2010 version)

X: NIMDM recorded; blank: NIMDM missing

**Table S2: Multivariable associations between social mobility class membership (2010-2016) and death (2017-2021)**

|  | **Hazard Ratio (95%CI)** |
| --- | --- |
| **Latent class** |  |
| Class 1: Stable high deprivation | 1.00 |
| Class 2: Moderate downward mobility | 0.87 (0.86,0.88) * |
| Class 3: Substantial downward mobility | 0.79 (0.78,0.79) * |
| Class 4: Stable medium deprivation | 0.80 (0.79,0.80) * |
| Class 5: Substantial upward mobility | 0.94 (0.94,0.95) * |
| Class 6: Moderate upward mobility | 0.76 (0.76,0.77) * |
| Class 7: Stable low deprivation | 0.65 (0.64,0.65) * |
| **Sex** |  |
| Male | 1 |
| Female | 0.88 (0.88,0.88) * |
| **Age (years) ^†^** |  |
| 0-17 | 1 |
| 18-34 | 2.62 (2.59,2.97) * |
| 35-54 | 12.56 (12.38, 12.73) * |
| 55+ | 129.71 (127.96,131.48) * |
| **Locale of residence** |  |
| Urban | 1 |
| Rural | 0.88 (0.82,0.83) * |

CI: Confidence Interval

*p<0.001

**^†^** Age categories 55-74 and 75 years plus amalgamated due to very high risk of death among oldest age-group

**Table S3: Multivariable associations between social mobility class membership (2010-16) and receipt of psychotropic medication (2017-21). Coefficients represent a) receipt (Odds Ratios) b) rate of receipt (Incidence Rate Ratios)**

|  | **Antidepressants** | **Anxiolytics ^†^** | **Hypnotics ^††^** |
| --- | --- | --- | --- |
| **Receipt of drug group** | **OR (95%CI)** | **OR (95%CI)** | **OR (95%CI)** |
| **Latent class** |  |  |  |
| Class 1: Stable high deprivation | 1 | 1 | 1 |
| Class 2: Moderate downward mobility | 0.85 (0.84,0.86) *** | 0.84 (0.83,0.85) *** | 0.87 (0.85,0.88) *** |
| Class 3: Substantial downward mobility | 0.78 (0.76,0.79) *** | 0.77 (0.75,0.78) *** | 0.79 (0.77,0.81) *** |
| Class 4: Stable medium deprivation | 0.75 (0.74,0.76) *** | 0.75 (0.74,0.77) *** | 0.75 (0.73,0.76) *** |
| Class 5: Substantial upward mobility | 0.87 (0.86,0.88) *** | 0.89 (0.87,0.91) *** | 0.88 (0.86,0.89) *** |
| Class 6: Moderate upward mobility | 0.69 (0.68,0.70) *** | 0.73 (0.72,0.74) *** | 0.69 (0.68,0.70) *** |
| Class 7: Stable low deprivation | 0.58 (0.58,0.59) *** | 0.62 (0.61,0.63) *** | 0.60 (0.59,0.61) *** |
| **Sex** |  |  |  |
| Male | 1 | 1 |  |
| Female | 2.05 (2.03,2.06) *** | 1.92 (1.90,1.94) *** | 1.45 (1.44,1.47) *** |
| **Age (years)** |  |  |  |
| 0-17 | 1 | 1 | 1 |
| 18-34 | 2.74 (2.71,2.77) *** | 3.64 (3.57,3.70) *** | 1.95 (1.92,1.99) *** |
| 35-54 | 4.01 (3.96,4.05) *** | 4.71 (4.63,4.79) *** | 3.14 (3.09,3.19) *** |
| 55-74 | 3.75 (3.70,3.80) *** | 5.25 (5.15,5.34) *** | 4.51 (4.43,4.59) *** |
| 75+ | 3.39 (3.32,3.46) *** | 6.45 (6.29,6.61) *** | 7.77 (7.59,7.96) *** |
| **Locale of residence** |  |  |  |
| Urban | 1 | 1 | 1 |
| Rural | 0.77 (0.77,0.78) *** | 0.84 (0.83,0.84) *** | 0.82 (0.81,0.83) |
|  |  |  |  |
| **Number of prescriptions per year among recipients** | **IRR (95%CI)** | **IRR (95%CI)** | **IRR (95%CI)** |
| **Latent class** |  |  |  |
| Class 1: Stable high deprivation | 1 | 1 | 1 |
| Class 2: Moderate downward mobility | 0.90 (0.89,0.91) *** | 0.90 (0.88,0.92) *** | 0.97 (0.95,0.99) |
| Class 3: Substantial downward mobility | 0.84 (0.83,0.85) *** | 0.82 (0.80,0.85) *** | 0.87 (0.84,0.90) |
| Class 4: Stable medium deprivation | 0.85 (0.84,0.86) *** | 0.83 (0.81,0.85) *** | 0.89 (0.87,0.92) |
| Class 5: Substantial upward mobility | 0.90 (0.89,0.92) *** | 0.91 (0.88,0.93) *** | 0.97 (0.94,1.00) |
| Class 6: Moderate upward mobility | 0.79 (0.78,0.80) *** | 0.78 (0.77,0.80) | 0.82 (0.80,0.84) |
| Class 7: Stable low deprivation | 0.69 (0.69,0.70) *** | 0.65 (0.63,0.66) | 0.69 (0.67,0.71) |
| **Sex** |  |  |  |
| Male | 1 | 1 | 1 |
| Female | 1.15 (1.14,1.16) *** | 1.02 (1.01,1.04) ** | 1.00 (0.99,1.02) |
| **Age (years)** |  |  |  |
| 0-17 | 1 | 1 | 1 |
| 18-34 | 1.67 (1.65,1.70) *** | 1.38 (1.34,1.43) *** | 0.75 (0.73,0.78) *** |
| 35-54 | 2.37 (2.34,2.40) *** | 1.74 (1.69,1.79) *** | 1.38 (1.35,1.42) *** |
| 55-74 | 2.15 (2.12,2.18) *** | 2.26 (2.19,2.30) *** | 2.19 (2.14,2.26) *** |
| 75+ | 2.17 (2.13,2.21) *** | 3.08 (2.96,3.19) *** | 3.16 (3.06,3.25) *** |
| **Locale of residence** |  |  |  |
| Urban | 1 | 1 | 1 |
| Rural | 0.93 (0.92,0.94) *** | 0.87 (0.86,0.88) *** | 0.98 (0.96,1.00) * |

OR: Odds Ratio; CI: Confidence Interval; IRR: Incidence Rate Ratio

*p<0.05, **p<0.01, ***p<0.001

^†^ capped at 12 prescriptions per person (2017-2021) for estimation purposes; ^††^ capped at 30 prescriptions per person (2017-2021) for estimation purposes

**Table S4: Multivariable associations between social mobility class membership (2010-2016) and presentations to A&E departments (2017-21) Coefficients represent a) attendance (Odds Ratios) b) rate of attendance (Incidence Rate Ratios)**

| **Attendance at A & E** | **OR (95%CI)** |
| --- | --- |
| **Latent class** |  |
| Class 1: Stable high deprivation | 1 |
| Class 2: Moderate downward mobility | 0.94 (0.93, 0.95) ** |
| Class 3: Substantial downward mobility | 0.89 (0.88, 0.91) ** |
| Class 4: Stable medium deprivation | 0.92 (0.91, 0.94) ** |
| Class 5: Substantial upward mobility | 1.01 (0.99, 1.02) |
| Class 6: Moderate upward mobility | 0.82 (0.81, 0.83) ** |
| Class 7: Stable low deprivation | 0.72 (0.72, 0.73) ** |
| **Sex** |  |
| Male | 1 |
| Female | 1.00 (0.99,1.00) |
| **Age (years)** |  |
| 0-17 | 1 |
| 18-34 | 0.80 (0.79,0.80) ** |
| 35-54 | 0.83 (0.82,0.84) ** |
| 55-74 | 1.31 (1.29,1.32) ** |
| 75+ | 3.25 (3.17,3.33) ** |
| **Locale of residence** |  |
| Urban | 1 |
| Rural | 1.00 (0.99,1.01) |
|  |  |
| **Number of visits per year at A & E among attenders** | **IRR (95%CI)** |
| **Latent class** |  |
| Class 1: Stable high deprivation | 1 |
| Class 2: Moderate downward mobility | 0.92 (0.90,0.93) ** |
| Class 3: Substantial downward mobility | 0.88 (0.86,0.89) ** |
| Class 4: Stable medium deprivation | 0.88 (0.86,0.89) ** |
| Class 5: Substantial upward mobility | 0.94 (0.93,0.96) ** |
| Class 6: Moderate upward mobility | 0.80 (0.79,0.81) ** |
| Class 7: Stable low deprivation | 0.71 (0.70,0.72) ** |
| **Sex** |  |
| Male | 1 |
| Female | 1.01 (1.00,1.02) * |
| **Age (years)** |  |
| 0-17 | 1 |
| 18-34 | 0.92 (0.91,0.93) ** |
| 35-54 | 0.97 (0.96,0.98) ** |
| 55-74 | 1.46 (1.45,1.48) ** |
| 75+ | 2.25 (2.41,2.48) ** |
| **Locale of residence** |  |
| Urban | 1 |
| Rural | 0.90 (0.89,0.90) ** |

A & E: Accident & Emergency; OR: Odds Ratio; CI: Confidence Interval; IRR: Incidence Rate Ratio;

*p<0.05; ***p<0.001

|  | | | **Hazard Ratios (95%CI)** | | | | | | | |  | |
| --- | --- | --- | --- | --- | --- | --- | --- | --- | --- | --- | --- | --- |
| **Null hypothesis** | **Alternative hypothesis** | **Rationale** | **Class 1:**  **Stable high deprivation** | **Class 2:**  **Moderate downward mobility** | **Class 3:**  **Substantial downward mobility** | **Class 4:**  **Stable medium deprivation** | **Class 5:**  **Substantial upward mobility** | **Class 6:**  **Moderate upward mobility** | **Class 7:**  **Stable low deprivation** | **p-value** | |  |
| a: The risk of death does not vary between the stable low deprivation class (Class 7), the stable medium deprivation class (Class 4) and the stable high deprivation class (Class 1) | a: The risk of death is lowest in the stable low deprivation class (Class 7), higher in the stable medium deprivation class (Class 4) and highest in the stable high deprivation class (Class 1) * | Higher levels of deprivation tend to be associated with poorer health outcomes | 1 |  |  | 0.80  (0.79,0.80) |  |  | 0.65 (0.64,0.65) | <0.001 | |  |
| b: The risk of death does not vary between the stable low deprivation class (Class 7), the substantial downward mobility class (Class 3) and the moderate downward mobility class (Class 2) | b: The risk of death is lowest in the stable low deprivation class (Class 7), higher in the substantial downward mobility class (Class 3) and highest in the moderate downward mobility class (Class 2) * | Levels of deprivation in the moderate downward mobility class (Class 2) remained consistently higher than the substantial downward mobility class (Class 3) |  | 0.87 (0.86,0.88) | 0.79 (0.78,0.79) |  |  |  | 0.65 (0.64,0.65) | <0.001 | |  |
| c: The risk of death does not vary between the stable high deprivation class (Class 1), the substantial upward mobility class (Class 5) and the moderate upward mobility class (Class 6) | c: The risk of death is highest in the stable high deprivation class (Class 1), lower in the substantial upward mobility class (Class 5) and lowest in the moderate upward mobility class (Class 6) * | Levels of deprivation in the substantial upward mobility class (Class 5) remained consistently higher than the moderate upward mobility class (Class 6) | 1 |  |  |  | 0.94 (0.94,0.95) | 0.76 (0.76,0.77) |  | <0.001 | |  |
| d: The risk of death does not differ between the substantial downward mobility class (Class 3), the stable medium deprivation class (Class 4) and the substantial upward mobility class (Class 5) | d: The risk of death differs between the substantial downward mobility class (Class 3), the stable medium deprivation class (Class 4) and the substantial upward mobility class (Class 5) ** | Health outcomes may not be a function of current deprivation but prior social mobility |  |  | 0.79 (0.78,0.79) | 0.80 (0.79,0.80) | 0.94 (0.94,0.95) |  |  | <0.001 | |  |

**Table S4 Post hoc hypothesis testing** **of the associations between social mobility class membership (2010-2016) and death (2017-2021)**

CI: Confidence Interval; * one-sided test for trend; ** two-sided test of inequality

**Table S5 Post hoc hypotheses testing of the associations between social mobility class membership (2010-2016) and receipt of antidepressants (2017-2021)**

|  | | | **Odds Ratios (95%CI) from hurdle models** | | | | | | | |  | |
| --- | --- | --- | --- | --- | --- | --- | --- | --- | --- | --- | --- | --- |
| **Null hypothesis** | **Alternative hypothesis** | **Rationale** | **Class 1:**  **Stable high deprivation** | **Class 2:**  **Moderate downward mobility** | **Class 3:**  **Substantial downward mobility** | **Class 4:**  **Stable medium deprivation** | **Class 5:**  **Substantial upward mobility** | **Class 6:**  **Moderate upward mobility** | **Class 7:**  **Stable low deprivation** | **p-value** | |  |
| a: Receipt of antidepressants does not vary between the stable low deprivation class (Class 7), the stable medium deprivation class (Class 4) and the stable high deprivation class (Class 1) | a: Receipt of antidepressants is lowest in the stable low deprivation class (Class 7), higher in the stable medium deprivation class (Class 4) and highest in the stable high deprivation class (Class 1) * | Higher levels of deprivation tend to be associated with poorer health outcomes | 1 |  |  | 0.75  (0.74,0.76) |  |  | 0.58 (0.58,0.59) | <0.001 | |  |
| b: Receipt of antidepressants does not vary between the stable low deprivation class (Class 7), the substantial downward mobility class (Class 3) and the moderate downward mobility class (Class 2) | b: Receipt of antidepressants is lowest in the stable low deprivation class (Class 7), higher in the substantial downward mobility class (Class 3) and highest in the moderate downward mobility class (Class 2) * | Levels of deprivation in the moderate downward mobility class (Class 2) remained consistently higher than the substantial downward mobility class (Class 3) |  | 0.85 (0.84,0.86) | 0.78 (0.76,0.79) |  |  |  | 0.58 (0.58,0.59) | <0.001 | |  |
| c: Receipt of antidepressants does not vary between the stable high deprivation class (Class 1), the substantial upward mobility class (Class 5) and the moderate upward mobility class (Class 6) | c: Receipt of antidepressants is highest in the stable high deprivation class (Class 1), lower in the substantial upward mobility class (Class 5) and lowest in the moderate upward mobility class (Class 6) * | Levels of deprivation in the substantial upward mobility class (Class 5) remained consistently higher than the moderate upward mobility class (Class 6) | 1 |  |  |  | 0.87 (0.86,0.88) | 0.69 (0.68,0.70) |  | <0.001 | |  |
| d: Receipt of antidepressants does not differ between the substantial downward mobility class (Class 3), the stable medium deprivation class (Class 4) and the substantial upward mobility class (Class 5) | d: Receipt of antidepressants does not differ between the substantial downward mobility class (Class 3), the stable medium deprivation class (Class 4) and the substantial upward mobility class (Class 5) ** | Health outcomes may not be a function of current social mobility but prior social mobility |  |  | 0.78 (0.76,0.79) | 0.75  (0.74,0.76) | 0.87 (0.86,0.88) |  |  | <0.001 | |  |

CI: Confidence Interval; * one-sided test for trend; ** two-sided test of inequality

**Table S6 Post hoc hypothesis testing of social mobility class membership (2010-2016) and A&E attendance (2017-2021)**

CI: Confidence Interval; A & E; Accident & Emergency; * one-sided test for trend; ** two-sided test of inequality

|  | | | **Odds Ratios (95%CI) from hurdle models** | | | | | | | |  | |
| --- | --- | --- | --- | --- | --- | --- | --- | --- | --- | --- | --- | --- |
| **Null hypothesis** | **Alternative hypothesis** | **Rationale** | **Class 1:**  **Stable high deprivation** | **Class 2:**  **Moderate downward mobility** | **Class 3:**  **Substantial downward mobility** | **Class 4:**  **Stable medium deprivation** | **Class 5:**  **Substantial upward mobility** | **Class 6:**  **Moderate upward mobility** | **Class 7:**  **Stable low deprivation** | **p-value** | |  |
| a: A&E attendance does not vary between the stable low deprivation class (Class 7), the stable medium deprivation class (Class 4) and the stable high deprivation class (Class 1) | a: A&E Attendance is lowest in the stable low deprivation class (Class 7), higher in the stable medium deprivation class (Class 4) and highest in the stable high deprivation class (Class 1) * | Higher levels of deprivation tend to be associated with poorer health outcomes | 1 |  |  | 0.92  (0.91, 0.94) |  |  | 0.72  (0.72, 0.73) | <0.001 | |  |
| b: A&E attendance does not vary between the stable low deprivation class (Class 7), the substantial downward mobility class (Class 3) and the moderate downward mobility class (Class 2) | b: A&E Attendance is lowest in the stable low deprivation class (Class 7), higher in the substantial downward mobility class (Class 3) and highest in the moderate downward mobility class (Class 2) * | Levels of deprivation in the moderate downward mobility class (Class 2) remained consistently higher than the substantial downward mobility class (Class 3) |  | 0.94  (0.93, 0.95) | 0.89  (0.88, 0.91) |  |  |  | 0.72  (0.72, 0.73) | <0.001 | |  |
| c: A&E attendance does not vary between the stable high deprivation class (Class 1), the substantial upward mobility class (Class 5) and the moderate upward mobility class (Class 6) | c: A&E Attendance is highest in the stable high deprivation class (Class 1), lower in the substantial upward mobility class (Class 5) and lowest in the moderate upward mobility class (Class 6) * | Levels of deprivation in the substantial upward mobility class (Class 5) remained consistently higher than the moderate upward mobility class (Class 6) | 1 |  |  |  | 1.01  (0.99, 1.02) | 0.82  (0.81,0.83) |  | <0.001 | |  |
| d1: A&E attendance does not differ between the substantial downward mobility class (Class 3), the stable medium deprivation class (Class 4) and the substantial upward mobility class (Class 5) | d: A&E Attendance does not differ between the substantial downward mobility class (Class 3), the stable medium deprivation class (Class 4) and the substantial upward mobility class (Class 5) ** | Health outcomes may not be a function of current social mobility but prior social mobility |  |  | 0.89  (0.88, 0.91) | 0.92  (0.91, 0.94) | 1.01  (0.99, 1.02) |  |  | <0.001 | |  |

**Fig S1 Multivariable associations between sociodemographic variables and outcomes (Odds Ratios)**

**Fig S2 Multivariable associations between sociodemographic variables and outcomes (Incidence Rate Ratios)**

**Fig S3 Estimated mean trajectories of area-level deprivation (2010-2016): 7 class Latent Class Growth Analysis (LCGA) solution (n=1,669,898)**

**References**

[1] D. Nagin, *Group-based modeling of development*, vol. 43, no. 02. Cambridge, MA: Harvard University Press, 2005. doi: 10.5860/choice.43-1258.

[2] B. O. Muthén and L. K. Muthén, “Integrating person-centered and variable-centered analyses: Growth mixture modeling with latent trajectory classes,” *Alcohol Clin Exp Res*, vol. 24, no. 6, 2000, doi: 10.1111/j.1530-0277.2000.tb02070.x.

[3] F. Chen, K. A. Bollen, P. Paxton, P. J. Curran, and J. B. Kirby, “Improper Solutions in Structural Equation Models,” *Sociol Methods Res*, vol. 29, no. 4, 2001, doi: 10.1177/0049124101029004003.

[4] K. A. S. Wickrama, T. K. Lee, C. W. O’Neal, and F. O. Lorenz, *Higher-Order Growth Curves and Mixture Modelling with Mplus*. New York, Oxford: Routledge, 2016.

[5] M. Rhemtulla, P. É. Brosseau-Liard, and V. Savalei, “When can categorical variables be treated as continuous? A comparison of robust continuous and categorical SEM estimation methods under suboptimal conditions,” *Psychol Methods*, vol. 17, no. 3, 2012, doi: 10.1037/a0029315.

[6] J. Benson and J. A. Fleishman, “The robustness of maximum likelihood and distribution-free estimators to non-normality in confirmatory factor analysis,” *Qual Quant*, vol. 28, no. 2, 1994, doi: 10.1007/BF01102757.

[7] Y. Lo, N. R. Mendell, and D. B. Rubin, “Testing the number of components in a normal mixture,” *Biometrika*, vol. 88, no. 3, pp. 767–778, Oct. 2001, doi: 10.1093/BIOMET/88.3.767.

[8] S. L. Clark, “Mixture Modeling with Behavioral Data,” University of California, Los Angeles, CA, 2010.

[9] G. J. McLachlan, S. X. Lee, and S. I. Rathnayake, “Finite mixture models,” *Annu Rev Stat Appl*, vol. 6, 2019, doi: 10.1146/annurev-statistics-031017-100325.

[10] C. Mack, Z. Su, and D. Westreich, “Managing Missing Data in Patient Registries: Addendum to Registries for Evaluating Patient Outcomes: A User’s Guide, Third Edition,” *Rockville, MD: Agency for Healthcare Research and Quality.*, 2018.

[11] C. Geiser, *Data Analysis with Mplus*. New York: The Guildford Press, 2013.
